# Transcutaneous auricular vagus nerve stimulation as an innovative approach for managing perioperative gastrointestinal dysfunction: a narrative review

**DOI:** 10.3389/fmed.2025.1719275

**Published:** 2025-12-03

**Authors:** Ling Zhang, Qin Zhang, Xuefeng Yin, Ya Cao, Ye Chen, Zhonghua Li, Yu Shen, Keyu Fan, Mingxia Liu, Lu Qian, Yunfeng Wang, Chongchun Dai, Yanjuan Ren, Jingqiu Wei, He Liu

**Affiliations:** 1 Key Laboratory of Anesthesia and Analgesia Application Technology, Department of Anesthesiology and Clinical Research Center for Anesthesia and Perioperative Medicine, Huzhou Central Hospital, The Fifth School of Clinical Medicine of Zhejiang Chinese Medical University, Huzhou, Zhejiang, China; 2 Huzhou Central Hospital, Affiliated Central Hospital Huzhou University, Huzhou, Zhejiang, China; 3 Affiliated Huzhou Hospital, Zhejiang University School of Medicine, Huzhou, Zhejiang, China; 4 Department of Education and Training, Huzhou Central Hospital, The Fifth School of Clinical Medicine of Zhejiang Chinese Medical University, Huzhou, Zhejiang, China; 5 Huzhou Key Laboratory of Basic Research and Clinical Translation for Neuromodulation, Huzhou Central Hospital, The Fifth School of Clinical Medicine of Zhejiang Chinese Medical University, Huzhou, Zhejiang, China

**Keywords:** postoperative gastrointestinal dysfunction, postoperative ileus, transcutaneous auricular vagus nerve stimulation, vagus nerve, pathophysiological mechanisms

## Abstract

Postoperative gastrointestinal dysfunction (POGD) is a prevalent and clinically significant complication following surgical procedures. It adversely impacts patient comfort, prolongs postoperative hospitalization duration, and increases the risk of perioperative complications and unplanned readmissions, thereby contributing to higher healthcare costs. Transcutaneous auricular vagus nerve stimulation (taVNS) has emerged as a promising neuromodulatory intervention. As a non-invasive technique, it offers several advantages, including ease of administration, a favorable safety profile, and high patient acceptability. This narrative review outlines the pathophysiological mechanisms underlying POGD, the anatomical basis of the vagus nerve, the technical parameters of taVNS, and its therapeutic mechanisms in managing postoperative gastrointestinal disturbances. The objective is to support the development of a comprehensive treatment approach for patients with POGD, promoting accelerated recovery.

## Introduction

Postoperative gastrointestinal dysfunction (POGD), commonly known as postoperative ileus (POI), is a common complication following surgical procedures. The incidence of POGD after abdominal surgery ranges from 10% to 30% (1). POGD is characterized by symptoms including nausea, vomiting, abdominal tenderness and distension, absence of normal bowel sounds, and delayed passage of flatus or stool (2). This condition adversely affects patient comfort, prolongs hospitalization, and increases the risk of perioperative complications and readmission, thereby contributing to elevated healthcare costs (3, 4). In recent years, various strategies have been developed to enhance postoperative gastrointestinal recovery, such as administration of alvimopan, adoption of minimally invasive surgical techniques, application of electroacupuncture stimulation (5), consumption of coffee-based beverages (6), intraoperative use of dexmedetomidine (7), chewing gum therapy, intravenous lidocaine infusion (8), and early initiation of enteral nutrition. Despite these advances, there remains a significant need for further improvement in the management of POGD. Therefore, it is essential to continue investigating effective interventions to reduce postoperative gastrointestinal impairment.

The primary objective of transcutaneous auricular vagus nerve stimulation (taVNS) is to provide a non-invasive therapeutic modality through electrical stimulation of the auricular branch of the vagus nerve (9). Over the past two decades, taVNS has evolved into a significant focus of investigation in basic science, clinical research, and translational medicine. It has increasingly been recognized as a viable alternative to pharmacological treatments for a range of disorders, including epilepsy, depression, cardiovascular conditions, and gastrointestinal diseases (10).

As a non-invasive neuromodulation technique, taVNS demonstrates significant therapeutic potential for POGD. This narrative review aims to clarify the pathophysiological mechanisms underlying POGD, the anatomical basis of the vagus nerve, the technical parameters of taVNS, and its therapeutic mechanisms in mitigating postoperative gastrointestinal complications. The primary objective is to provide a comprehensive reference for clinical application and to support the integration of taVNS into standardized postoperative rehabilitation protocols.

## Methods

A comprehensive literature search was conducted in the PubMed database to identify English-language articles without restrictions on publication year. The search strategy was based on a combination of keywords, including “vagus nerve stimulation,” “transcutaneous auricular vagus nerve stimulation,” “postoperative gastrointestinal dysfunction,” and “postoperative ileus.” All study designs were eligible for inclusion in this review, such as systematic reviews, randomized controlled trials, descriptive and analytical studies, and narrative reviews.

## Pathophysiological mechanisms underlying POGD

The etiology, duration, and severity of POGD are not yet fully understood. These factors are intricately associated with multiple physiological mechanisms, including spinal-gut sympathetic reflexes, sympathetic hyperactivity, inflammatory mediators, opioid administration, electrolyte disturbances, as well as anesthetic and surgical techniques. During laparotomy and abdominal surgery, activation of the sympathetic pathway and inhibitory non-adrenergic vagally mediated pathways has been consistently observed, contributing to gastrointestinal hypomotility intraoperatively and in the immediate postoperative period (11). Surgical incision of the abdominal wall and entry into the peritoneal cavity stimulate spinal afferent fibers, which synapse in the spinal cord and initiate an inhibitory cascade involving prevertebral adrenergic neurons, ultimately suppressing gastrointestinal motility (12). Furthermore, intense visceral afferent stimulation during manipulation of the intestinal loops activates supraspinal centers, leading to the recruitment of hypothalamic and pontine-medullary nuclei (12). This results in the engagement of an inhibitory non-adrenergic pathway via the vagus nerve, which reduces gut motility through the release of nitric oxide (NO) and vasoactive intestinal peptide (VIP) (11).

The involvement of inflammatory mechanisms plays a critical role in the sustained and clinically significant impairment of gastrointestinal motility (13). Following surgical trauma and intestinal manipulation, the outer muscular layer mounts an inflammatory response mediated by a diverse array of immune cells, including macrophages, dendritic cells, mast cells, monocytes, and neutrophils (2). Among these, muscularis macrophages serve as key regulators of gastrointestinal homeostasis and are critically involved in the pathogenesis of motility disorders (14). Surgical manipulation activates muscularis macrophages, triggering an intracellular inflammatory cascade characterized by the upregulation of multiple transcription factors—such as NF-κB, STAT3, p38-MAPK, and early growth response protein 1—which subsequently drive the expression of pro-inflammatory genes (15–17). Consequently, the release of chemokines and cytokines—such as interleukin (IL)-1, monocyte chemoattractant protein-1 (MCP-1), IL-6, and tumor necrosis factor-α (TNF-α)—as well as kinetically active substances including NO and prostaglandins, is triggered (15, 18–20). During intestinal surgery, mast cells undergo degranulation and release mediators that promote local infiltration within the intestinal wall, thereby disrupting normal intestinal motility (21). Furthermore, reactive intestinal glial cells, neurons, smooth muscle cells, enteroendocrine cells, epithelial cells, and the microbiota present in the intestinal lumen collectively contribute to the pathogenesis of POGD. Enteric glial cells play a critical role in modulating neural circuit activity within the enteric nervous system—often referred to as the guts “little brain”—and are essential for maintaining normal motility. Surgical stress, excessive mechanical manipulation, ischemia/reperfusion injury, infections, and bacterial toxins can induce the transformation of enteric glial cells into a reactive phenotype, resulting in enteric gliosis. These glial responses contribute to neuroinflammation and enteric neuropathy, which clinically manifest as impaired motility, diarrhea, increased intestinal permeability, and abdominal or visceral pain (22).

The gut microbiota plays a crucial role in maintaining intestinal homeostasis. Exposure to surgical stressors—such as anesthesia, perioperative antibiotic administration, and invasive surgical procedures—induces significant alterations in the composition and functional activity of the gut microbial community. In particular, severe physiological trauma has been shown to markedly affect both the abundance and metabolic functionality of the gut microbiota. Gastrointestinal surgery increases intestinal permeability and impairs the integrity of the intestinal barrier, thereby promoting dysbiosis and facilitating bacterial translocation (23). The gut microbiota and its metabolites modulate intestinal motility through multiple mechanisms: (1) induction of dendritic cells to produce IL-12, which activates T helper type 1 (Th1) cells to secrete interferons (IFNs), subsequently stimulating mast cells and macrophages to release pro-inflammatory mediators such as NO, TNF, and IL-1β; (2) inhibition of tryptophan hydroxylase 1 (TPH1)-mediated synthesis of 5-hydroxytryptamine (5-HT) in enterochromaffin cells, where 5-HT regulates the release of neurotransmitters including substance P; (3) direct suppression of neuronal surface receptors, such as Toll-like receptor (TLR)2 and TLR4; and (4) inhibition of neuronal nuclear receptors, such as the aryl hydrocarbon receptor (AHR), leading to reduced transcription of key neurotransmitter-related genes (24).

## Anatomical basis of the auricular branch of the vagus nerve

The vagus nerve, the tenth cranial nerve, is the longest and most extensively distributed of all cranial nerves. As a mixed nerve, it consists of approximately 20% efferent fibers and 80% afferent fibers, thereby integrating both sensory and motor functions (25). Afferent sensory fibers convey somatic and visceral sensory information to the brainstem, while efferent visceral motor fibers modulate neurogenic, myogenic, and endocrine activities in target organs^25^. The vagus nerve mediates bidirectional communication between the central nervous system and multiple major anatomical structures, including the pharynx, larynx, trachea, heart, aorta, lungs, and the entire gastrointestinal tract—encompassing the esophagus, stomach, liver, pancreas, and spleen (26).

The anatomical structure of the external ear consists primarily of eight components: the helix, antihelix, triangular fossa, scapha, concha (divided into the cavum conchae and cymba conchae), tragus, antitragus, and the earlobe (27). Of particular note, the concha is the only superficial region where the auricular branch of the vagus nerve is distributed, rendering it an ideal site for transcutaneous electrical stimulation (28, 29). Human functional magnetic resonance imaging (fMRI) studies have shown that stimulation of the auricular branch of the vagus nerve induces significant activation in the “classical” central vagal projection areas, including the nucleus tractus solitarius (NTS), locus coeruleus (LC), spinal trigeminal nucleus (STN), parabrachial nucleus, dorsal raphe, periaqueductal gray, amygdala, insula, nucleus accumbens, bed nucleus of the stria terminalis, and paracentral lobule (30). In contrast, decreased activity has been observed in the hippocampus and hypothalamus (30). The dorsal motor nucleus (DMN) functions as the primary efferent center for central regulation of visceral parasympathetic activity and receives direct input from the NTS (31). These centrally integrated signals are subsequently relayed to peripheral organs innervated by the vagus nerve, leading to the release of neurotransmitters and modulation of multiple organ functions (27).

## Transcutaneous auricular vagus nerve stimulation

taVNS is an innovative, non-invasive neuromodulation technique that targets the auricular branch of the vagus nerve. Compared with invasive vagus nerve stimulation, this method is characterized by its favorable safety profile and high tolerability, with only mild and transient adverse effects—such as otalgia, headache, and paresthesia—reported in the literature (32). In the ongoing discussion regarding the optimal stimulation target for taVNS, current research primarily focuses on safety and efficacy (32). A systematic review reported an overall adverse event rate of 3.6% (20 out of 562 participants), with the most common events including ear discomfort, adhesive allergy, tinnitus, and insomnia (33). Given the traditional understanding that the efferent fibers of the vagus nerve innervating the heart are predominantly located on the right side, left-ear-only taVNS is generally considered safe in most studies. Nevertheless, evidence suggests that bilateral or right-sided stimulation does not lead to significant adverse effects (34). Stimulation parameters—such as optimal intensity, pulse width, waveform, and frequency—may vary across different populations and require further investigation.

## Potential mechanisms of taVNS on POGD

**FIGURE 1:**
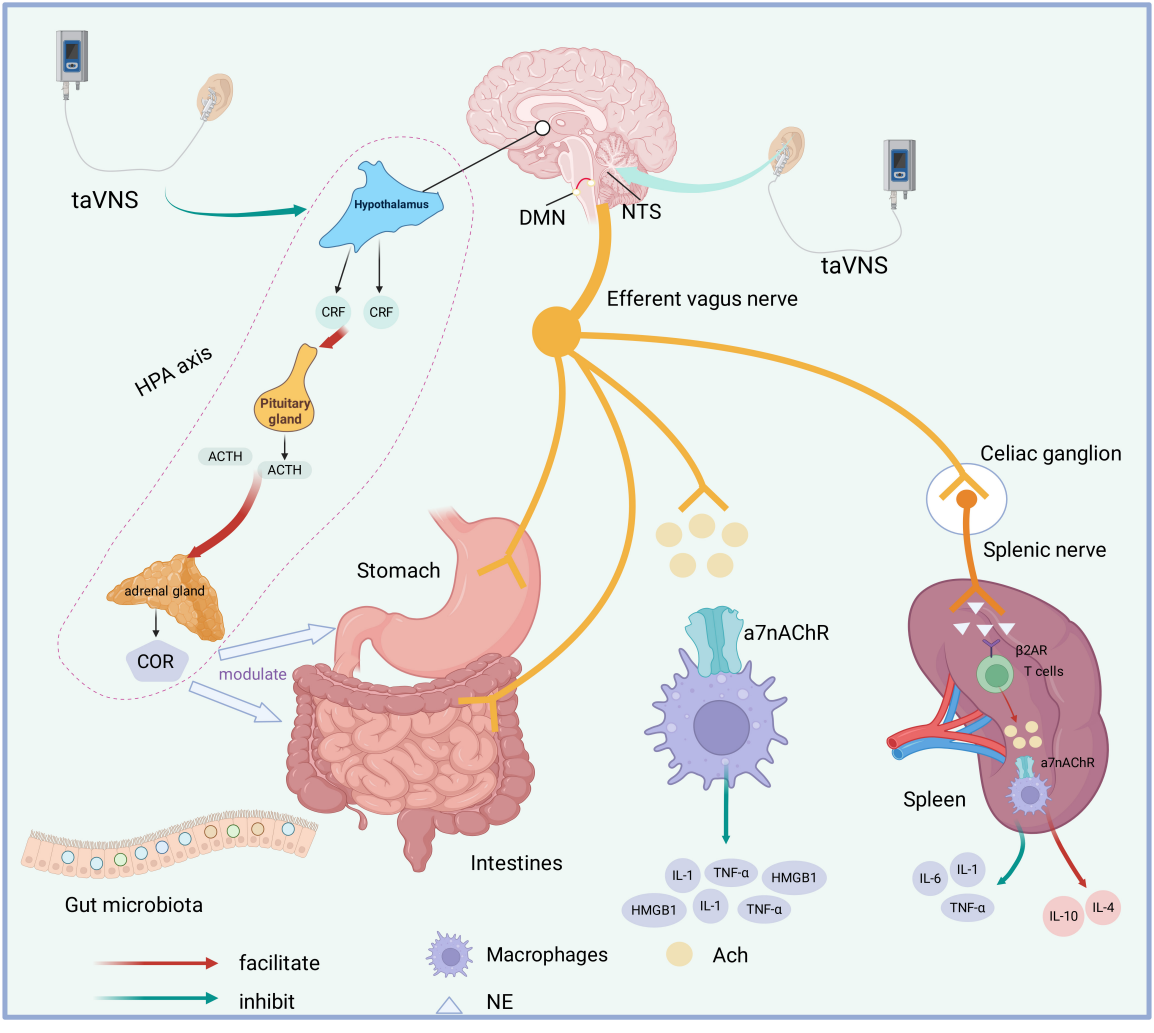
Potential mechanisms of taVNS on POGD. TaVNS induces activation of NTS and DMN. Through the efferent pathway of the vagus nerve, it may directly modulate gastrointestinal motility and secretion, improve intestinal microbiota composition, and activate both the cholinergic anti-inflammatory pathway and splenic nerve afferent pathways. TaVNS inhibits HPA axis hyperactivity. DMN, dorsal motor nucleus; NTS, nucleus tractus solitarius; taVNS, transcutaneous auricular vagus nerve stimulation; POGD, postoperative gastrointestinal dysfunction; α7nAChR, nicotinic acetylcholine receptor α7 subunit; β2AR, β2-adrenergic receptor; ACh, acetylcholine; IL, interleukin; NE, norepinephrine; TNF-α, tumor necrosis factor-α; CRH, corticotropin-releasing factor; ACTH, adrenocorticotropic hormone; HPA axis, hypothalamic–pituitary–adrenal axis; HMGB1, high mobility group box 1;COR, cortisol.

taVNS may improve POGD through multiple mechanisms, including activation of the vagus nerve, modulation of inflammatory responses, and regulation of neuroendocrine activity and gut microbiota (Figure 1).

### taVNS facilitates gastrointestinal regulation

taVNS delivers electrical stimulation to the auricular branches of the vagus nerve, thereby activating afferent fibers. The generated signal is transmitted via the NTS to the DMN of the vagus nerve, leading to enhanced activity of efferent pathways. This mechanism significantly increases parasympathetic tone and promotes gastrointestinal motility and secretory function. In a study conducted on healthy volunteers, cold stimulation was shown to reduce the percentage of normal gastric slow waves and suppress vagal activity; however, this impairment was subsequently ameliorated by taVNS treatment (35). taVNS has been demonstrated to enhance vagal activity, improve gastric accommodation, and increase the proportion of normal gastric slow waves in patients with functional dyspepsia (FD) (36). Furthermore, non-invasive auricular vagus nerve stimulation has been found to accelerate colonic and whole-gut transit times and restore enteric neural function through central and vagal efferent pathways, effectively alleviating constipation symptoms in a rodent model of opioid-induced constipation (OIC) (37).

### taVNS modulates neuroendocrine function

The hypothalamic-pituitary-adrenal (HPA) axis constitutes a central component of the neuroendocrine system, essential for regulating stress responses, immune function, and metabolic homeostasis. Under stressful conditions, such as those experienced during abdominal surgery and associated pain, corticotropin-releasing factor (CRF) neurons—predominantly located in the parvocellular region of the paraventricular nucleus (PVN)—are activated (38). This activation leads to the release of CRF, which stimulates the anterior pituitary gland to secrete adrenocorticotropic hormone (ACTH). In turn, ACTH promotes cortisol production in the adrenal cortex and facilitates adrenaline release from the adrenal medulla (39). Furthermore, CRF signaling pathways in both the central nervous system and the gastrointestinal tract are critically involved in mediating stress-induced changes in gastrointestinal motility (40). Intravenous administration of CRF has been demonstrated to induce both paracellular and transcellular intestinal barrier dysfunction. taVNS has been shown to reduce paracellular permeability in the small intestine, thereby improving intestinal barrier integrity. Furthermore, taVNS effectively suppresses hyperactivity of the HPA axis (41). Elevated cortisol levels are associated with alterations in gut microbiota composition and increased intestinal permeability, which may promote systemic inflammation and contribute to central nervous system dysfunction and related disorders (42). A study investigating the antidepressant-like effects of three different frequencies of taVNS in rats subjected to chronic unpredictable mild stress (CUMS) revealed that 20 Hz taVNS significantly reduced plasma corticosterone and ACTH levels, leading to downregulation of HPA axis activity (43). In a FD rat model, taVNS has also been found to attenuate HPA axis hyperactivation (44).

### taVNS modulates immune function

In 2002, Tracey introduced the concept of the cholinergic anti-inflammatory pathway (CAP), a mechanism through which the parasympathetic nervous system modulates inflammatory responses via efferent vagus nerve signaling (45). Activation of the CAP is primarily mediated by the interaction between acetylcholine (ACh) and α7 nicotinic acetylcholine receptors (α7nAChR) expressed on macrophages (46), leading to the suppression of pro-inflammatory cytokine release, includingTNF-α, IL-1β, and high mobility group box 1 (HMGB1). Although the vagus nerve does not innervate gut-resident macrophages directly, it communicates with enteric neurons expressing neuronal nitric oxide synthase (nNOS), VIP, and choline acetyltransferase (ChAT), which are located in the muscularis externa in close proximity to α7nAChR-expressing macrophages (47). In a murine model of lipopolysaccharide (LPS)-induced endotoxemia, taVNS at 15 Hz was shown to attenuate inflammation by activating α7nAChR and reducing TNF-α and IL-1β levels (48). Moreover, taVNS has been demonstrated to alleviate oxidative stress and inhibit mitochondrial apoptosis in a 1,2-dimethylhydrazine (DMH)-induced colon cancer model (49). Furthermore, taVNS upregulates the CAP, as evidenced by increased expression of α7nAChR and decreased expression of nuclear factor kappa B p65 (NF-κB p65), TNF-α, and HMGB1 at both protein and mRNA levels (49).

A substantial body of research has consistently demonstrated that neural stimulation activates splenic sympathetic nerve fibers, resulting in the release of norepinephrine. This neurotransmitter subsequently binds to β2-adrenergic receptors on acetylcholine-producing T cells, thereby promoting the secretion of Ach (50). The released ACh then interacts with α7nAChR expressed on splenic macrophages, leading to effective suppression of TNF-α production by these immune cells.

### taVNS alleviates gut microbiota dysbiosis

The human gut microbiota harbors trillions of microorganisms that play a critical role in modulating both local and systemic immune responses (51). In the context of ischemic stroke, taVNS has been demonstrated to attenuate the systemic inflammatory response following cerebral ischemia-reperfusion injury. Furthermore, taVNS ameliorates gut microbiota dysbiosis and strengthens the integrity of both the blood-brain barrier (BBB) and intestinal barrier by regulating intestinal microbial imbalances and inhibiting mast cell degranulation, thereby reducing translocation of LPS from the gut (21). In a murine model of constipation-predominant irritable bowel syndrome (IBS-C), taVNS significantly decreased the abdominal withdrawal reflex (AWR) score relative to sham stimulation, indicating effective alleviation of visceral hyperalgesia (52). Additionally, taVNS restored the abundance of Lactobacillus and increased that of *Bifidobacterium* at the genus level. It also enhanced the population of c-kit-positive interstitial cells of Cajal (ICC) in the myenteric plexus region in IBS-C mice (53). A single-center randomized controlled trial on the effects of taVNS in patients with IBS-C demonstrated significant increases in *Bifidobacterium* levels and concentrations of short-chain fatty acids (SCFAs), including acetic, butyric, and propionic acids, following four weeks of treatment. Furthermore, taVNS was associated with reduced levels of tryptophan-derived metabolites, such as 3-hydroxyanthranilic acid and L-tryptophan (53). *Bifidobacterium* has been shown to upregulate the activity of suppressive regulatory T cells, maintain intestinal barrier integrity, modulate dendritic cell and macrophage functions, and attenuate intestinal Th2 and Th17 immune responses (54).

## taVNS for the treatment of gastrointestinal dysfunction

In recent years, numerous clinical trials have demonstrated the efficacy of taVNS in treating gastrointestinal dysfunction, as summarized in Table 1. Recent studies demonstrate that taVNS effectively improves gastrointestinal motility and symptoms across disorders such as FD, IBS-C, and POI. By enhancing vagal efferent activity and restoring gut–brain axis balance, taVNS accelerates recovery, normalizes gastric rhythms, and alleviates discomfort (36, 53, 55, 56). Importantly, all studies reported excellent safety, with no serious adverse events and only mild, transient local discomfort. Collectively, these findings support taVNS as a clinically meaningful and well-tolerated intervention capable of improving gastrointestinal function through modulation of autonomic and enteric neural pathways, warranting further large-scale, long-term trials to confirm its therapeutic efficacy and optimize stimulation parameters.

**TABLE 1:** Summary of clinical trials on transcutaneous auricular vagus nerve stimulation (taVNS) for gastrointestinal dysfunction.

References	Model	Sample size and justification	Stimulation parameter	Duration	Study design	Control group	Primary and secondary outcomes	Adverse events
Huang et al. (55)	Patients undergoing gastrointestinal surgery	*n* = 22 (11 taVNS, 11 sham); sample size calculation was not reported.	No report.	15 min daily until first flatus	RCT	Sham stimulation	↓Time to first flatus (62 ± 21 h vs. 90 ± 27 h), ↑gastric motility complexity and rate	None reported
Ru et al. (56)	Patients who received scheduled laparoscopic radical resection of colorectal cancer	*n* = 148; powered to detect POI reduction from 20% → 6.25% (α = 0.05, power 0.8, taking into account the possibility of patients being eliminated from the trial)	25 Hz, 50 mA, 20 min, right auricular branch	Single session before anesthesia	RCT	No-stimulation control	↓POI incidence (6.25% vs. 20%), faster bowel sound recovery (24–48 h)	None reported
Zhu et al. (36)	Patients with functional dyspepsia	*n* = 36 functional dyspepsia patients (18 taVNS, 18 sham) + 39 healthy controls; power analysis based on previous data (80% power, α = 0.05)	25 Hz, 0.5 ms, 0.5–1.5 mA, 2 s on/3 s off, bilateral cymba concha	1 h twice/day × 2 weeks	RCT	Sham-ES at non-vagal arm site	↓Dyspepsia, anxiety, depression; ↑gastric accommodation, ↑% normal gastric slow waves, ↑vagal tone	Mild (1 in taVNS) all manageable
Liu et al. (53)	Patients with constipation-predominant irritable bowel syndrome	*n* = 22,G*Power based on VAS pain difference (power 80%, α = 0.05, allowing 20% dropout)	25 Hz, 0.5 ms pulse width, 0–2 mA (max tolerated), 2 s on/3 s off, unilateral auricular concha	30 min/day × 4 weeks	RCT	Sham-taVNS at earlobe and antihelix	↓VAS, ↓IBS-SSS, ↑bowel frequency, ↑Ach, ↓NO, ↑HRV-HF; improved rectal sensitivity and microbiome balance	Mild (3 in taVNS, 2 in control), all manageable

## Other methods for managing POGD

### Electroacupuncture

Electroacupuncture is a contemporary clinical therapeutic approach that integrates traditional acupuncture techniques with the precise delivery of electrical stimulation, allowing for standardized and quantifiable control of stimulation parameters such as frequency and intensity. Electroacupuncture has been shown to effectively reduce postoperative nausea and vomiting, accelerate the time to first flatus and defecation, and alleviate postoperative gastrointestinal dysfunction (5, 57, 58). Electroacupuncture is invasive and involves risks such as local pain, bleeding and infection. Due to the differences in acupuncture culture, its application and promotion in other countries have been restricted.

### Dexmedetomidine

Dexmedetomidine, a selective agonist of alpha2-adrenergic receptors, is often employed as an anesthesia adjunct during the perioperative stage. This is because it possesses anti-inflammatory characteristics, offers stress-reducing advantages, and exerts a beneficial impact on the vagus nerve. A multicenter clinical trial the administration of intraoperative dexmedetomidine reduced the time to first flatus, time to first feces, and length of stay after abdominal surgery without affecting the I-FEED score (7). The adverse effects of dexmedetomidine are primarily confined to hemodynamic alterations, including bradycardia, transient hypertension, and hypotension. There was no significant difference in these adverse events between the 2 groups (7). A systematic review and meta-analysis suggested that perioperative administration of dexmedetomidine can facilitate the recovery of gastrointestinal function and shorten the length of hospital stay for patients after abdominal surgery (59). However, the optimal dose and timing of dexmedetomidine need further investigation.

### Chewing gum

Chewing gum can stimulate the vagus nerve in the head and neck region, promote gastrointestinal motility, and reduce the transmission of sympathetic neural signals (60). Concurrently, it enhances the secretion of saliva and pancreatic juice (60). Multiple meta-analysis results have shown that chewing gum can reduce postoperative nausea and vomiting, shorten the time for the first defecation and flatus, and accelerate the recovery of gastrointestinal function (60–62). Using chewing gum as an auxiliary measure in routine postoperative care to promote the recovery of the gastrointestinal tract after surgery requires more high-quality clinical research.

### Intravenous lidocaine

Lidocaine is a local anesthetic agent known for its anti-inflammatory and tissue-protective properties. It has been administered intravenously as an adjunct in general anesthesia across various surgical procedures, demonstrating efficacy in reducing early postoperative pain scores and decreasing opioid consumption. Some single-center randomized controlled trials have shown that intravenous administration of lidocaine during the perioperative period can lead to faster recovery of intestinal function, reduced pain, and shorter hospital stay (63–65). However, the results of a multicenter randomized study showed that perioperative administration of lidocaine did not improve the recovery of intestinal function within 72 h (8). Based on this, the current evidence does not support the use of conventional intravenous lidocaine to accelerate postoperative gastrointestinal recovery.

### Coffee-based beverage

Following laparoscopic gynecological surgery, coffee consumption was associated with a reduced time to first flatus, earlier time to first bowel movement, and shorter time to tolerance of solid food, thereby promoting the recovery of gastrointestinal function (66). During a cesarean section, the consumption of coffee can also facilitate the recovery of gastrointestinal functions (67). A meta-analysis of randomized controlled trials revealed that consuming coffee following surgery was associated with a reduced time to first bowel movement after elective colorectal procedures (68). However, the optimal dosage of coffee, whether it contains caffeine or not, as well as the impact of other chemical components in coffee on the recovery of gastrointestinal function still require further exploration.

### Alvimopan

In 2008, the U.S. Food and Drug Administration approved alvimopan, an oral, peripherally acting opioid μ-receptor antagonist, for the acceleration of gastrointestinal recovery in patients undergoing intestinal resection surgery (69). Alvimopan has the most consistent benefit for open bowel resections but is costly and not universally indicated.

## Discussion and conclusion

### Current limitations in clinical application

At present, the application of taVNS in POGD is still in its early exploratory stages. Although taVNS has shown potential therapeutic value for gastrointestinal dysfunction, its clinical application faces several challenges. Firstly, Mechanistic clarity vs clinical translation. Although there is a satisfactory explanation for the vagus nerve regulation at the mechanism level, the conversion of these physiological changes into meaningful clinical outcomes still requires validation in large-scale surgical samples. Second, the evidence of the research and the quality issues. Published studies are few, typically small, and heterogeneous in terms of surgical populations, the timing of stimulation, and co-interventions. Third, the scope of the research subjects is rather limited. The majority of existing studies concentrate on elective abdominal procedures among relatively healthy adult populations. However, there remains a paucity of evidence regarding high-risk patient groups—such as individuals with frailty or severe cardiopulmonary comorbidities—as well as emergency surgeries and non-abdominal operations where POGD still occurs, including spinal surgeries. Fourth, within the framework of the Enhanced Recovery After Surgery (ERAS) program, interventions such as early enteral feeding, multimodal analgesia, regional anesthesia, and early mobilization have been shown to significantly influence gastrointestinal function recovery. Inconsistent application or inadequate reporting of these components may confound the assessment of the independent effect of taVNS. Fifth, safety characterization and device considerations. Reported adverse events are usually mild (local irritation, lightheadedness), but data in patients with implanted cardiac devices, significant bradyarrhythmias, or skin disorders around the ear are limited.

### Feasibility in perioperative settings

Applying taVNS in the perioperative context holds promise but also presents practical considerations:

1. Timing of intervention: There is a question of when to apply stimulation — pre-operative, intraoperative, early postoperative.
2. Integration with standard perioperative care: taVNS should be incorporated into established analgesic protocols, ERAS pathways, and routine patient monitoring procedures. It is essential to provide healthcare personnel with comprehensive training on proper electrode placement—targeting the auricular branch of the vagus nerve—and on the ongoing assessment of device functionality to ensure treatment efficacy and patient safety.
3. Device logistics and patient state: During equipment transportation and patient management, post-operative patients may present with dressings, attached monitoring leads, or symptoms such as pain or sedation. These factors can influence the placement of ear electrodes, as well as patient compliance and comfort. Additionally, it is essential to monitor for stimulation-related adverse reactions, including bradycardia and hypotension.
4. Workflow and cost-effectiveness: To achieve the promotion, this intervention measure must not excessively prolong the work flow during the surgery, nor require expensive equipment.
5. Patient selection: Identifying which patients (type of surgery, risk of POGD) are most likely to benefit is key.

### Standardization challenges

Standardized treatment protocols are lacking. key stimulation parameters—such as frequency, intensity, and duration—have not been systematically established, warranting rigorous optimization to improve therapeutic outcomes.

Frequency: In the existing literature, the stimulation frequencies employed in taVNS exhibit considerable variability, ranging from 1 to 30 HZ. To establish a standardized frequency range for clinical application, further research is required, as variations in frequency may significantly influence treatment efficacy and patient tolerance.

Intensity: Numerous studies employ the “perceivable yet comfortable” paradigm, calibrating stimulation intensity according to individual sensory thresholds. However, inter-individual variability among patients and fluctuations during the treatment process may influence the consistency of this approach. Consequently, there is a need for a more standardized methodology to measure and adjust stimulation intensity, ensuring reproducible outcomes while minimizing discomfort or overstimulation.

Duration: The duration of sessions and the timing of stimulation also differ between studies. There is no relevant literature available that specifies the appropriate timing or duration for applying the stimulus.

## Conclusion

As a non-invasive neuromodulation technique, taVNS has shown promising potential in the treatment of gastrointestinal dysfunction. It may improve postoperative gastrointestinal function through multiple mechanisms, including activation of the vagus nerve, modulation of inflammatory responses, and regulation of neuroendocrine activity and gut microbiota. However, existing research remains preliminary, and further exploration of its underlying mechanisms, as well as well-designed, high-quality clinical trials, is required to support its broader clinical implementation.
